# Responding to the global cholera pandemic

**DOI:** 10.2471/BLT.23.020423

**Published:** 2023-04-01

## Abstract

An increase in cholera outbreaks is prompting renewed calls for urgent multisectoral action. Fid Thomson reports.

Christophe Valingot has watched the recent spread of cholera around the world with an increasing sense of frustration. “Cholera has been preventable and treatable for decades and yet here we are in the midst of an accelerating global pandemic,” he says. “It shouldn’t be happening.”

An epidemiologist and water sanitation engineer who consults with the International Federation of Red Cross and Red Crescent Societies (IFRC) on their global cholera response, Valingot has been on the front lines in many emergencies. “Each one is different, but the challenges are always the same, starting with lack of access to safe water and sanitation,” he says.

The cholera pandemic to which Valingot refers is the seventh, as documented by epidemiologists. Driven almost exclusively by the *Vibrio cholerae* biotype El Tor, serogroup O1, the pandemic started in 1961 and tailed off in 1975. Since then, it has continued to smoulder, with an estimated 2.9 million cases and 95 000 deaths per year (the true disease burden is unknown, because the majority of cases are not reported) and has recently flared up in multiple hotspots.

“By the end of 2022, 30 countries worldwide were battling cholera outbreaks, some experiencing their largest epidemics in recent decades,” says Dr Natalie Fischer, an epidemiologist working on global outbreak monitoring in the World Health Organization’s (WHO) Health Emergencies Programme.

Rising case fatality is a major concern. Untreated, inappropriately treated, or treated too late, cholera can kill a person in a matter of hours and is particularly dangerous for children under the age of 5 years and children who are malnourished.

“In 2021, the average reported cholera case fatality rate (CFR) reached 1.9% and as high as 2.9% in Africa,” Fischer explains. “That is significantly higher than the less than 1%, which is expected with an adequate public health response. Preliminary data suggests the trend will continue in 2022 and 2023.”

Given that El Tor has not become significantly more pathogenic since the 1960s, the question arises: what is driving these trends?

For Fischer, among the likely drivers, climate-change-related emergencies loom large, notably those involving flooding and drought, both of which have implications for water, sanitation and hygiene. 

On the flooding front, Mozambique offers a prime example. The country has seen a sharp increase in cholera cases since December 2022 and, as of 5 March 2023, a cumulative total of 7517 suspected cases and 41 deaths (CFR 0.6%) had been reported in 33 districts of six of the country’s 11 provinces.

“Resources are being stretched very thin.”

Natalie Fischer

“All six provinces are flood-prone, and as the rainy season continues, it is anticipated that more districts will be affected,” says Adam Garley, Mozambique country director of the nongovernmental organization WaterAid. Cyclone Freddy, the longest-lived and strongest tropical cyclone on record, slammed into Mozambique on 24 February and again on 12 March, compounding the challenges faced.

Neighbouring Malawi is also struggling with a major, protracted cholera outbreak which started in Machinga district following tropical storm Ana and Cyclone Gombe in January and March 2022. Those events led to floods which displaced communities with low pre-existing immunity and lacking access to safe water and adequate sanitation. As of 5 March 2023, a total of 50 523 cases, including 1578 deaths, had been reported from all 29 districts in the country (CFR 3.1%) with active transmission ongoing in 25 of them.

Increasing levels of conflict are also contributing to cholera’s spread and lethality by forcing communities to relocate to places where accessing safe water and sanitation is a challenge, and often with limited access to health care. “Medical facilities are more likely to be depleted of stocks and personnel,” explains Valingot, “while people are less likely to travel in search of care when fighting is going on.”

In Haiti, the cholera outbreak first reported in October 2022 is spreading against a backdrop of political and economic instability, gang violence, and fuel shortages. This combination of factors has impacted access to both health care and safe water and sanitation and, as of 28 February, a cumulative total of 33 661 and 94 deaths had been reported (CFR 1.8%) from all 10 departments in the country.

Conflict on a much larger scale has impacted communities in the Syrian Arab Republic where, as of 28 February, 92 649 suspected cases of cholera had been reported, half in the northwest of the country. The risk of cholera was exacerbated by the powerful earthquakes that hit neighbouring southern Türkiye and northern Syrian Arab Republic on 6 February, disrupting water and sanitation infrastructure and health care already degraded by years of conflict.

In a statement released on 8 March, WHO warned of increased risk of waterborne diseases for over 2.1 million Syrians living in the northwest of the country, especially those living in overcrowded camps.

There are indications that the Syrian epidemic is also spilling into its neighbour Lebanon, which in 2022 reported its first case of cholera since 1993.

For Fischer, the sheer number of outbreaks is in some instances overwhelming response capacity, which in turn feeds into accelerated cholera transmission and mortality. “Resources are being stretched very thin,” she says. “This is reducing health systems’ capacity to inform, prevent, diagnose and treat, which inevitably impacts transmission and mortality.”

That being the case, it is crucial that countries make optimal use of the resources available to them, starting with prompt implementation of information campaigns and urgent care. As noted above, cholera can kill within hours, but it can be effectively and inexpensively treated with oral rehydration solution, supplemented with antibiotics and intravenous fluids for more severe cases.

“It would be difficult to overstate the importance of widespread implementation of ORT (oral rehydration therapy) at community level,” says Valingot, who explains that the IFRC is currently supporting its implementation in multiple countries, making sure that communities have the skills and capacity to provide it. “Oral rehydration solution is simple enough to mix, but the knowledge and skills required to deliver it can easily be lost, especially in countries that have not experienced cholera for many years,” he says.

Valingot also stresses the importance of simple awareness-raising about the disease. “Most of the deaths are happening because of lack of awareness that cholera can kill in hours,” he says. “People need to be made aware of this.”

“Most of the deaths are happening because of lack of awareness.”

Christophe Valingot

Pierre-Yves Oger, a water, sanitation and hygiene expert specializing in public health emergencies at the United Nations Children’s Fund, also emphasises the importance of community engagement. “We need to put communities at the centre of emergency response, providing them with the information they need about the risks of cholera and how households can protect themselves, for example by using protected water sources, washing hands, and boiling water.”

Dr Aninda Rahman, an epidemiologist at the Centers for Disease Control and Prevention in Dhaka, Bangladesh, underlines the fact that effective outbreak response includes their prompt declaration, noting that there is a tendency for governments to delay the reporting of cholera outbreaks due to concerns about negative economic consequences, and shame regarding the presence of the disease in their populations. “There is still a great deal of stigma attached to the disease,” he says, “a perception that it is a disease of backward, dirty people that should not have a place in the modern world.”

As Rahman points out, prompt declaration is not only crucial to awareness-raising but is required to access the oral cholera vaccines procured and distributed as part of international emergency response arrangements. These include access to the global oral cholera vaccine stockpile that grew to 35 million doses in 2022.

While assessed to be less than half of the estimated global annual demand of 75 million doses, the stockpile can still be a boon to countries in need of urgent support. “In 2022, Bangladesh was quick to declare the major cholera outbreak which ran from March to May, and was able to implement a vaccination campaign which reached 2.36 million people,” Rahman explains.

Ensuring access to safe water and sanitation in the midst of a crisis is central to effective emergency response, but permanent, sustainable access is needed to make progress against cholera over the long term. “Addressing the water, sanitation and hygiene challenge is clearly core to any sustainable solution for cholera,” says Valingot.

Oger could not agree more. Using data published by the WHO-hosted Global Task Force on Cholera Control (GTFCC), he recently completed a study of the relationship between poor water, sanitation and hygiene conditions and cholera cases between 2010 and 2021. “Almost all (96.7%) of the roughly 5 million cases reported to the GTFCC came from 31 countries that are among those ranked lowest for service access for water and sanitation,” he explains.

It is widely accepted that addressing the water, sanitation and hygiene problem will require a major multisectoral effort that will only be possible with high-level political support and investment. “Governments need to prioritize water, sanitation and hygiene services improvement through their national development plans but also through their budget allocation,” says Valingot. “Donors have an important role to play, but the governments in cholera-affected countries have to step up.”

**Figure:**
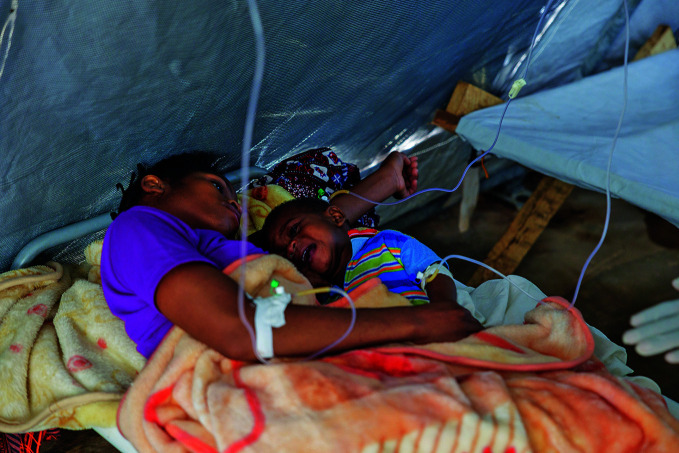
Mother and child receive intravenous fluids at a cholera treatment centre in Lichinga city, Niassa Province, Mozambique.

UNICEF/Ricardo Franco

**Figure:**
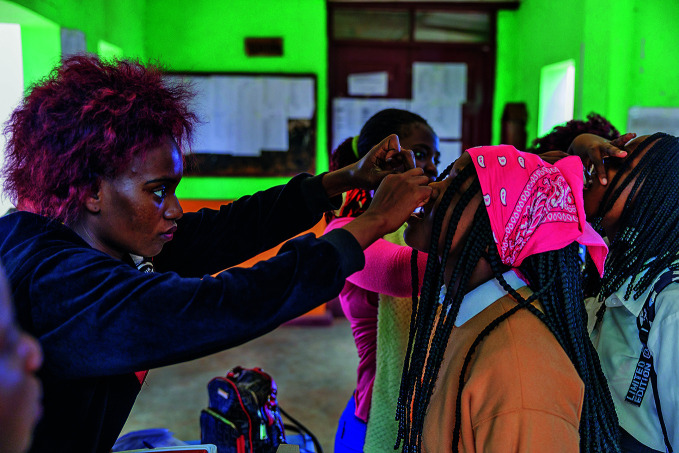
Oral cholera vaccine administered in a school in Lichinga city, Niassa Province, Mozambique.

UNICEF/Ricardo Franco

